# Experiences of environmental services workers in a tertiary hospital in Asia during the COVID-19 pandemic: a qualitative study

**DOI:** 10.3389/fpubh.2023.1178054

**Published:** 2023-06-05

**Authors:** Qin Xiang Ng, Nicholas Ye Kai Koh, Xiaohui Xin, Humairah Zainal, Jack Thian Tan, Julian Thumboo, Kok Yong Fong

**Affiliations:** ^1^Singapore General Hospital, Singapore, Singapore; ^2^NUS Yong Loo Lin School of Medicine, National University of Singapore, Singapore, Singapore; ^3^Duke-NUS Medical School, National University of Singapore, Singapore, Singapore

**Keywords:** COVID-19, environmental services workers, qualitative study, pandemic, work

## Abstract

**Background:**

The Coronavirus Disease 2019 (COVID-19) pandemic has had a significant impact on all walks of life, in particular, environmental services workers in healthcare settings had higher workload, increased stress and greater susceptibility to COVID-19 infections during the pandemic. Despite extensive literature describing the impact of the pandemic on healthcare workers such as doctors and nurses, studies on the lived experiences of environmental services workers in healthcare settings are sparse and none has been conducted in the Asian context. This qualitative study thus aimed to examine the experiences of those who worked for a year of the COVID-19 pandemic.

**Methods:**

A purposive sample of environmental services workers was recruited from a major tertiary hospital in Singapore. Semi-structured interviews were conducted in-person, lasting around 30min, and included open-ended questions pertaining to five main domains: work experiences during COVID-19, training and education needs, resource and supplies availability, communication with management and other healthcare staff, and perceived stressors and support. These domains were identified based on team discussions and literature review. The interviews were recorded and transcribed for thematic analysis, as guided by Braun and Clarke.

**Results:**

A total of 12 environmental services workers were interviewed. After the first seven interviews, no new themes emerged but an additional five interviews were done to ensure data saturation. The analysis yielded three main themes and nine subthemes, including (1) practical and health concerns, (2) coping and resilience, and (3) occupational adaptations during the pandemic. Many expressed confidence in the preventive efficacy of proper PPE, infection control practice and COVID-19 vaccination in protecting them against COVID-19 and severe illness. Having prior experience with infectious disease outbreaks and previous training in infection control and prevention appeared to be useful as well for these workers. Despite the various challenges presented by the pandemic, they could still find meaning in their everyday work by positively impacting the wellbeing of patients and other healthcare workers in the hospital.

**Conclusion:**

Besides uncovering the concerns shared by these workers, we identified helpful coping strategies, resilience factors and certain occupational adaptations, which have implications for future pandemic planning and readiness.

## 1. Introduction

The Coronavirus Disease 2019 (COVID-19) pandemic has had a significant impact on all walks of life (1). In particular, environmental services workers in healthcare settings who are responsible for cleaning and maintaining the cleanliness, hygiene and infection control of hospitals have been affected by the pandemic (2–4). In response to the pandemic, hospitals worldwide have implemented various information and training programs for these employees to ensure appropriate surface disinfection and cleaning (5), and enhanced terminal cleaning (ultraviolet light (UV-C), electrostatic spraying, or room fogging) after a COVID-19 patient is moved out of a room has also been adopted in some centers (6). Some of the impacts highlighted by previous studies include increased workload, added work stress and the heightened risk of exposure to the severe acute respiratory syndrome coronavirus 2 (SARS-CoV-2) virus (2–4). Skin-related problems (e.g., dryness, rash, and dermatitis) due to the use of personal protective equipment (PPE) are also common (7). However, it is concerning that in a study conducted in a tertiary care hospital in India, the risk of SARS-CoV-2-specific IgG seropositivity among housekeeping staff was found to be four times higher than doctors and nurses (8).

Although COVID-19 is a mild illness for most individuals, there are several reviews that suggest post COVID-19 condition as an emerging issue in occupational medicine, with workers who suffer persistent symptoms (such as fatigue, shortness of breath, and cognitive dysfunction) and have reduced quality of life and productivity (9, 10). The effects of COVID-19 on workers should be evaluated over time, especially for workers who have residual functional limitations and may require modified work duties or hours to facilitate their return to employment. Remote work is not an option for these frontline workers, who have been overloaded since the start of the COVID-19 pandemic.

Social epidemiology research has also found that COVID-19 accentuated the fault lines of socioeconomic and health inequality in many countries, with a disproportionate burden of disease and a greater likelihood of income loss and emotional distress among low-income communities (11–13). Based on the findings of earlier studies, compared to other frontline healthcare workers, environmental services workers have experienced similar (if not greater) levels of depression, anxiety, and stress due to increased job demands and the risk of COVID-19 exposure (2–4). These challenges include work-related difficulties during the pandemic, unmet training and education needs, lack of resources and supplies, poor communication with management and other healthcare staff, as well as other perceived stressors and lack of support (2–4). However, in-depth studies on their lived experiences during the pandemic are sparse (4) and none has been conducted in the Asian context.

Despite a significant body of literature describing the impact of the COVID-19 pandemic on healthcare workers such as doctors and nurses (14–17), limited attention has been paid to environmental services workers who are essential frontline workers and integral to the smooth functioning of a hospital. In contrast to other healthcare workers, environmental services workers are often contracted from external sources and may perceive themselves as invisible to the rest of the hospital team (18). Hence, this qualitative study aimed to provide new insights to the experiences of environmental services workers who worked during a year of the pandemic in a major tertiary hospital in Asia. The results of this study may serve as a basis for policy and procedural changes aimed at improving the status of environmental services workers, a group that is often overlooked in the broader healthcare workforce.

## 2. Methods

### 2.1. Study setting

This study was conducted in the largest tertiary hospital in Singapore, the Singapore General Hospital (SGH). SGH operates 2,000 inpatient beds, multiple specialist centers and outpatient clinics and caters to more than 1 million patients yearly (19). During the COVID-19 pandemic, like other hospitals in the rest of the world, SGH also faced a huge surge in patients with fever and respiratory symptoms seeking care at the Emergency Department, and dedicated wards were set up to care for COVID-19 patients (20). Staff also had to be redeployed into the community with the rises in community cases of COVID-19 with only mild symptoms.

### 2.2. Study participants

Participants for the study were recruited through nomination by the operations manager and by putting up posters on notice boards around the hospital, flyers left in staff common areas. Environmental services workers who have worked in SGH during the period of the COVID-19 pandemic from January 2021 to January 2022 were eligible to take part in the study. From those who were identified as eligible and were willing to participate in the study, a purposive sample was chosen to ensure a variety of staff of different profiles (in terms of age, gender and nationality) and who have worked in areas with close contact with COVID patients.

### 2.3. Procedures

In accordance with the four qualitative research traditions described by Creswell (21), we adopted a phenomenological methodology to capture the personal experiences of this specific group of workers. The interviews were conducted in-person by two study investigators (Q.X.N. and N.Y.K.K.) using a semi-structured interview guide, developed with the study team’s expertise and with reference to a prior qualitative study conducted in the United States (4). The interviews lasted around 30min and included open-ended questions pertaining to five main domains relevant to environmental services employees: work experiences during COVID-19, training and education needs, resource and supplies availability, communication with management and other healthcare staff, and perceived stressors and support. These domains were identified based on team discussions and literature review (2–4). The full interview guide can be found in online Supplementary material. The interviews were conducted in either English or Mandarin, where the respondents were conversant and had basic or higher proficiency of the language.

Interview recordings were then transcribed by removing elements of speech like pauses and non-verbal communication between the researcher and the interviewee. Where both English and Mandarin were used in data collection, only the Mandarin portions of the interaction was translated. All participants were allocated a unique code number in the transcripts to remain anonymous. All interview transcripts were cross-checked for accuracy before uploading to NVivo software (v12) for coding.

In order to ensure the credibility of the data, we utilized two methods. The first was “member checks” (22), which involved comparing our interpretation of the data with that of the participants to ensure consistency, either at the end of an interview or just prior to the next one. The second was “reflexivity” (23), where the interviewers carefully examined their research experience, possible biases, and preconceptions before each data collection.

### 2.4. Data analysis

Data was analysed using a thematic analysis approach, as guided by Braun and Clarke (24), to identify sub-themes and themes. Thematic analysis was chosen as it has theoretical flexibility, provides a detailed and nuanced analysis, and is useful for studying individual experiences, opinions, and views (25). By reading and re-reading the transcripts, the study investigators familiarized themselves with the data, produced preliminary codes, formulated overarching themes, reviewed and refined themes, defined and specified themes, and produced a write-up (24). Study investigators reviewed the transcripts independently, and coding disagreements were resolved by further discussion until consensus was reached. Throughout the process, the data analysis was shared and discussed continuously between all the authors. The study investigators also moved back and forth between the different steps during the analysis in an iterative manner.

Ethical approval for the study was obtained from the SingHealth Centralised Institutional Review Board (CIRB 2022/2629). Participants were provided with a participant information sheet explaining the rationale and details of the study before signing an informed consent form prior to the start of the interview.

## 3. Results

A total of 12 staff were interviewed. After the first seven interviews, no new themes emerged but an additional five interviews were done to ensure data saturation was reached. The participants’ demographics are listed in Table 1; majority were females (*n* = 7, 58.3%), and the median age of the participants was 32.5 (range 26–70) years, with a median work experience of 4.5 (range 3–22) years. There was a diversity of participants with different nationalities, with three participants hailing from Malaysia and Singapore, and two participants each from Myanmar, Philippines and the People’s Republic of China, respectively.

**Table 1:** Demographics of study participants.

Profile	Number of participants
**Gender**	
Male	5
Female	7
**Age range**	
21–30 years	4
31–40 years	3
41–50 years	2
51–60 years	1
61–70 years	2
**Nationality**	
Malaysia	3
Myanmar	2
People’s Republic of China	2
Philippines	2
Singapore	3
**Years of experience working at SGH**	
1–5 years	7
6–10 years	3
11–15 years	1
16–20 years	0
21–25 years	1

**Figure 1:**
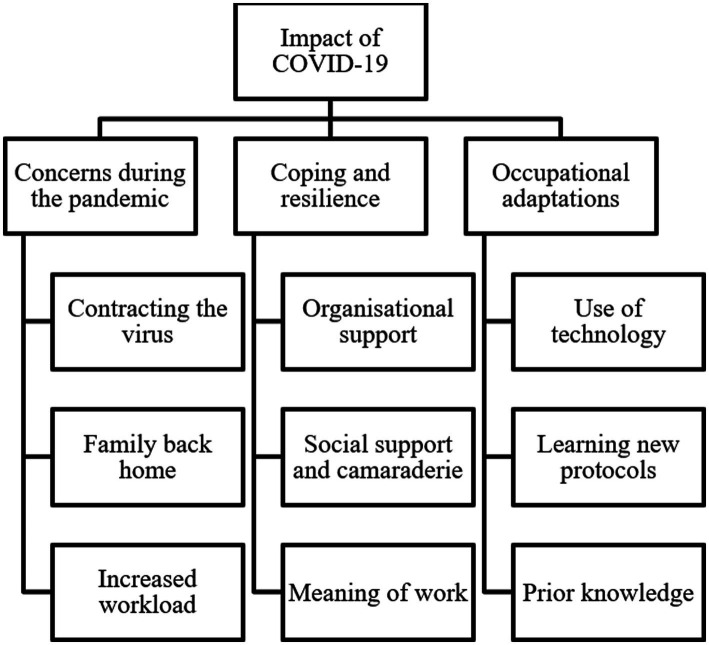
Thematic map of themes and subthemes identified.

Three themes and nine subthemes are reported alongside interview verbatim quotes (Table 2) and a thematic map (Figure 1). The themes and subthemes are explained in the following table.

**Table 2:** Themes and subthemes from thematic analysis, with accompanying sample quotations.

Themes and subthemes	Quotations
**Theme 1: concerns during COVID-19 pandemic**	
Contracting SARS-CoV-2 virus	“Honestly, when I found out the company sent me to cover the covid ward, honestly I wanted to resign. I mean all of the world at that time scared of COVID so why must I sacrifice myself? I am the sole breadwinner for my family so if something happens to me, how?” (P2) “To be honest at the start when we were unvaccinated, it was really quite frightful stepping into the rooms of COVID positive patients. You really feel directly at risk and exposed to the virus with certain vulnerabilities.” (P6)
Worry about family back home	“Because I come from Myanmar right, so I worry about family back home since I cannot go home and can only call my mom.” (P1) “As a Malaysian, with the border closures, I used to go home more regularly like at least a few times a month but I did not expect that the border remained closed for so long. So I really missed my family and had to try to keep myself motivated to keep working here.” (P12)
Increased workload	“It was frankly quite hectic because there were a lot of changes and new protocols that I had to get used to. The patient load was higher as well and we had to wear PPE including safety goggles when doing our work.” (P8) “There were more protocols so the cleaning itself took longer and we had to do double or triple job because of manpower shortage. And we had to wear the PPE all the time, it was very hot and difficult to work in the PPE.” (P11)
**Theme 2: coping and resilience during COVID-19 pandemic**	
Organizational support	“So we had this ‘Heroes Day’ in the hospital and we were celebrated as well. This really helped me feel a part of the healthcare team.” (P5) “The hospital gave us free food, NTUC vouchers and provided showering facilities and a change of clothes after we end work daily. This was definitely a big improvement.” (P8)
Social support and camaraderie among healthcare team	“The hospital leaders, doctors and nurses all treat us very respectfully and they really took the effort to take care of our welfare by taking care of our physical needs.” (P8) “And after working in the ward for a few weeks the doctors and nurses will remember me and can chit chat a bit, like “how are you?,” “everything OK?,” “patients keep coming and coming right,” some small jokes and laughing. Although not so talkative during COIVD but I really feel a part of the team with them.” (P12)
Connecting meaning to work	“I received a card from a primary school kid that wrote, “not every angel has wings, some wear PPE.” I thought that was really very touching and made me feel proud to work in a hospital even if I am just doing housekeeping.” (P11) “I feel like as a housekeeper I also have a needed set of skills. I can clean the rooms properly. When the patients are discharged, they would also tell us things like ‘Thank you brother’. So that really gives me meaning.” (P12)
**Theme 3: occupational adaptations**	
Benefits of technology for work	“I think it could reduce manual labor needs, like the ecobot can automatically move and clean by itself. And you also do not need an extra person to supervise the cleaning that way.” (P4) “The newer UV-C models could clean the entire room in a shorter duration of time and they were less bulky.” (P6)
Learning new protocols	“We had a lot of changes to our workflow. We had to wear N95, monitor and report our temperatures daily and communicate with our team if we develop any flu symptoms. For myself I did not really feel it was a challenge because I was flexible to these changes.” (P10) “The hospital managers and our supervisors all came down to train us to make sure we know how to wear the PPE properly. So that was quite good.” (P11)
Tapping on prior knowledge	“I had prior experience working with infectious diseases patients and also received training during H1N1, so it was easier for me.” (P4) “I was a nurse actually back in my home country so I think I am quite used to outbreak situations.” (P10)

### 3.1. Theme 1: concerns during the COVID-19 pandemic

#### 3.1.1. Subtheme 1a: contracting the SARS-CoV-2 virus

Several respondents expressed concerns about working during the pandemic, especially being exposed to COVID-19 patients and their environments. However, the perceived susceptibility to the virus was alleviated with personal protective equipment (PPE) use and COVID-19 vaccination, “For me I felt very lucky to have the vaccines, like very early on they offered us the chance to take the vaccines. There was no fear of catching COVID even when I was on the frontline of receiving COVID patients.” (P5).

For respondents who were infected and came down with COVID-19, interestingly, they expressed newfound confidence and strength after recovering from COVID-19, “Like I said, at first was catching COVID because you do not know what the virus can do to your body. But after I caught it and recovered, after that quite OK because you know how to deal with it already. That’s the thing we have to face and live with. If you are infected, you just need to isolate yourself and try to be healthy again.” (P12).

#### 3.1.2. Subtheme 1b: worry about family back home

As a number of the employees were foreign workers, they expressed worries about the health, safety and status of their families back home, especially since they were unable to visit them with several countries closing their border to nonessential travel during the pandemic. “My family in the Philippines were all infected by COVID last year and they were not vaccinated, so I was quite worried about them.” (P5).

#### 3.1.3. Subtheme 1c: increased workload

Predictably, due to increased protocols, work demands and manpower shortages during the pandemic, several respondents expressed concerns about the increase in workload during the pandemic period. “More detailed cleaning also. Will take almost 2 h to clean the room after the patient is discharged.” (P2).

### 3.2. Theme 2: coping and resilience during the pandemic

#### 3.2.1. Subtheme 2a: organizational support

Based on the sentiments of several respondents, small gestures made by the organization helped them feel appreciated and kept them going during the pandemic. “The hospital gave us extra allowance, extra leave, free food and cards with notes of appreciation. All these things were really nice and made me feel supported.” (P4).

#### 3.2.2. Subtheme 2b: social support and camaraderie among healthcare professionals

Most of the interviewees also felt supported and part of the larger healthcare team and this sense of camaraderie was motivating. “The ward sisters [nurses] always appreciate us. They do not see us lower or higher. Always very nice to us and will offer us food and help.” (P2).

#### 3.2.3. Subtheme 2c: connecting meaning to work

Several respondents also saw their jobs as meaningful and felt cleaning to be a pivotal part of the hospital and patient care process. “So I will mop and clean harder and to me that may help patients get better … Because the patients also very sad and very stressed right. So long inside one place, cannot go out. So they will talk to us, “hey why you want to work this COVID? You not scared ah? You do not want to work other things?” And I think by talking to them, their burden and stress dropped. Makes me a bit proud of myself.” (P2).

### 3.3. Theme 3: occupational adaptations

#### 3.3.1. Subtheme 3a: benefits of technology for work

Some of the respondents were exposed to new cleaning technologies during the pandemic, and they felt that these automated technologies could help make their jobs easier and may provide additional sanitisation. “For our cleaning jobs, I think technology like the UV-C machine also provides additional sanitisation to the areas we are cleaning.” (P5).

However, at the same time, some respondents did express certain reservations about the use of technology for cleaning, “I think if we work with people rather than machines, more satisfied with the cleaning. Like teamwork like that.” (P2).

#### 3.3.2. Subtheme 3b: learning new protocols

In response to COVID-19, there were several new workflows and protocols and the employees had to adapt and learn these protocols to do their work well. “Some people were really scared to work with COVID patients, and got many new workflows and the work demands were stricter during COVID-19, so I think not everyone may be able to handle the job.” (P6).

#### 3.3.3. Subtheme 3c: tapping on prior knowledge

Some of the respondents expressed greater self-assurance when it came to COVID-19 as they had prior infection control and prevention training or had worked in the hospital during the earlier local H1N1 influenza outbreaks. “I think the experience during H1N1 helped. I felt that if I wore my PPE properly and followed the prescribed protocols, I know I am protected against the infection.” (P4).

## 4. Discussion

The analysis performed in this study yielded three main themes surrounding the lived experiences of environmental services workers working in a major tertiary hospital in Asia during a year of the COVID-19 pandemic. These include (1) practical and health concerns, (2) coping and resilience, and (3) occupational adaptations during the pandemic. The pandemic has been a significant stressor for health systems worldwide as it greatly increased the job demands for frontline healthcare workers (2–4, 14–17), and environmental services workers were also subjected to an increase in workload, as highlighted by the respondents in our study. Another added source of worry and stress for many of these migrant workers were the inability to visit their families due to the border closures and travel restrictions enacted by several countries during the pandemic (26).

Based on an earlier scoping review, environmental services workers appeared to be a particularly susceptible group to COVID-19 infections during the pandemic (2). Seroprevalence studies conducted elsewhere have highlighted higher rates of COVID-19 infections among environmental services workers (housekeeping, cleaning and janitorial staff) compared to other clinical and non-clinical staff (8, 27, 28). The respondents in our study did rightly share some of these concerns of working in the frontlines and being exposed to COVID-19 positive patients and their artifacts and surroundings. Nonetheless, many expressed confidence in the preventive efficacy of proper PPE, infection control practice and COVID-19 vaccination in protecting them against COVID-19 and severe illness. The workers appeared to be particularly receptive to COVID-19 vaccination. Singapore boasts a high vaccination coverage among the general public (29), and similar to the findings of a large global survey, trust in science and vaccine confidence are comparably stronger in countries with high social consensus (30). Having prior experience with infectious disease outbreaks and previous training in infection control and prevention appeared to be helpful as well for these workers, and this has important implications for future pandemic planning and preparedness.

Although several studies have been conducted on migrant occupational health around the world, the literature has used diverse definitions and varied psychometric measures (31), and a previous systematic review on the occurrence of common mental health issues among migrant workers worldwide has found significant variation in prevalence rates (32). Contributory and influencing factors include the age of the worker, the presence of health issues, individual coping skills, occupational factors (working conditions, salary and benefits etc.), environmental (living condition, access to healthcare etc.) and presence of social support (32). For example, the prevalence of depression among Myanmar migrant workers in Malaysia was as high as 70.8%, while the prevalence of depression among migrant workers in China was only 24.3% (32). While none of the respondents in the present study reported significant distress during the pandemic, there are several initiatives health systems and individuals can do to help environmental services workers during the COVID-19 pandemic. According to the findings of a previous qualitative study, environmental services workers may feel a lack of recognition as frontline workers as they are forgotten in mainstream media and hospital hierarchies impeded their day-to-day interactions with other members of the healthcare team (4). In contrast to other hospital staff, these workers are also often hired from external sources and may feel a lack of belonging to the hospital they work in. It is evident that even small gestures like food and handwritten notes temper these relations and motivate these workers, and employers, policymakers and other members of the healthcare team should continue to recognize the important work that they do. Mutual acknowledgment and collaboration would foster camaraderie among the healthcare team, and public recognition, incentives, and other forms of appreciation are all possible options to show appreciation for the contributions made environmental services workers. The lack of human connectivity was also felt by environmental services workers as they are probably used to working in teams and having breaks together. Given their susceptibility to COVID-19 infections, they should also be entitled to paid sick leave benefits to stay home if feeling unwell. In addition, to aid their cleaning, automated technologies like autonomous cleaning robots may be helpful (33).

Importantly, in our study, environmental services workers could find meaning in their everyday work as they took pride in positively impacting the health and wellbeing of staff and patients in the hospital despite the challenges presented by the pandemic. Similar to the findings of an earlier study (4), environmental services workers in the hospital recounted how even before the pandemic, interacting with patients and other members of the healthcare team brought them joy. As all the respondents were still working in the hospital at the point of the study, this may represent a positive deviance (34), but it nonetheless emphasizes the importance of meaning of work in order to keep employees engaged and motivated.

It is also worth mentioning that these workers were employed and supplied by a third-party contractor to work in the hospital. Worldwide, many hospitals have outsourced environmental services work to contractors, with associated issues such as inadequate training, protocol lapses and higher turnover observed (18). The insights gleaned from the present study suggest that the workers do not view themselves as “external” to the hospital they work in, and it is possible to engage them by investing in their wellbeing.

Limitations of the present study included the small number of employees in a single hospital setting. We were also unable to contact employees who resigned or left the hospital during the pandemic and as such, the workers most affected by the pandemic may have been left out in the study, and the views may not be representative of all workers during the pandemic. As Singapore is a relatively well-resourced country and has one of the lowest case fatality rates for COVID-19 in the world (35), the findings may also lack generalizability to other countries, organizational characteristics and cultures.

## 5. Conclusion

This is the first study to gain qualitative insights into the lived experience of environmental services workers working in a major tertiary hospital in Asia. Besides uncovering the concerns shared by these workers, we identified coping strategies, resilience factors and certain occupational adaptations relevant to environmental service work, which have important implications for future pandemic planning and readiness. Our findings also have significant implications for healthcare policy, as environmental services workers are often overlooked within the broader healthcare workforce. Consequently, we propose that this study may serve as a catalyst for targeted policy and procedural changes aimed at improving the status of environmental services workers within healthcare organizations.

## Data availability statement

The raw data supporting the conclusions of this article will be made available by the authors, without undue reservation.

## Ethics statement

The studies involving human participants were reviewed and approved by SingHealth Centralised Institutional Review Board (CIRB 2022/2629). The patients/participants provided their written informed consent to participate in this study.

## Author contributions

JTh and KF conceived the original idea. QN and NK carried out the study, investigation, and the relevant data analysis and interpretation. XX, HZ, JTa, KF, and JTh contributed to the data analysis and interpretation. KF and JTh supervised the study. All authors contributed to the writing and proofreading of the final manuscript. The final manuscript was approved by all authors. All authors agree to be accountable for the content of the work.

## Funding

This study and the article processing charge were supported by the SingHealth Duke-NUS Medicine Academic Clinical Programme under the Seah Cheng Siang Distinguished Professorship in Medicine.

## Conflict of interest

The authors declare that the research was conducted in the absence of any commercial or financial relationships that could be construed as a potential conflict of interest.

## Publisher’s note

All claims expressed in this article are solely those of the authors and do not necessarily represent those of their affiliated organizations, or those of the publisher, the editors and the reviewers. Any product that may be evaluated in this article, or claim that may be made by its manufacturer, is not guaranteed or endorsed by the publisher.
