# Supplementary material for: Experiences of environmental services workers in a tertiary hospital in Asia during the COVID-19 pandemic: a qualitative study
Source: Front Public Health. 2023 Jun 5;11:1178054. doi: 10.3389/fpubh.2023.1178054 (PMC10277473; doi:10.3389/fpubh.2023.1178054)
Supplement: Supplementary file 1 [file Table_1.DOCX]

Supplementary Material

Experiences of Environmental Services Workers in A Tertiary Hospital in Asia During the COVID-19 Pandemic: A Qualitative Study

Qin Xiang Ng, Nicholas Ye Kai Koh, Xiaohui Xin, Humairah Zainal, Jack Thian Tan, Julian Thumboo, and Kok Yong Fong*

*** Correspondence:** Prof Kok Yong Fong, fong.kok.yong@singhealth.com.sg

# Supplementary Data

Supplementary Material should be uploaded separately on submission. Please include any supplementary data, figures and/or tables.

Supplementary material is not typeset so please ensure that all information is clearly presented, the appropriate caption is included in the file and not in the manuscript, and that the style conforms to the rest of the article.

# Supplementary Figures and Tables

**Table S1.** Interview guide

**1. Work Experiences During COVID-19**

What has it been like working during COVID-19?

What has been a challenge about working during this time?

**2. Training and Education**

Did you feel sufficiently equipped to work during this time?

What was missing in how you were prepared?

Did you have apprehension about working with COVID-19-positive patients/rooms?

**3. Resources and Supplies**

Have you used any new cleaning supplies or equipment during COVID-19?

Has anything improved or worsened?

Has there been anything you needed that you could not get?

Do you think technology could make your jobs easier, why or why not?

**4. Communication**

How has the communication from leadership been for you when it comes to COVID-19?

How has communication been with other teammates, like doctors, pharmacists, social workers etc.?

What is missing from the communication/what issues have come up during this time?

**5. Stresses and Support**

How much do you feel a part of the larger healthcare team? In what ways?

What pressures have you felt during the COVID-19 response? How different were these from usual?

What are your biggest concerns when performing your work during COVID-19?

What has been set up to help support you during this time; how have you felt supported?

**6. Closing questions**

What do you wish could be different about how the hospital is working through the COVID-19 pandemic?

What gives you meaning in your work?

Is there anything else you'd like to add?
